# Clinical and biochemical characterization of a patient with prolidase deficiency, a rare disorder of collagen metabolism

**DOI:** 10.1016/j.ymgmr.2025.101258

**Published:** 2025-09-23

**Authors:** Troy K. Coody, Irene De Biase, Julie M. Porter, Marzia Pasquali, Brian J. Shayota

**Affiliations:** a Department of Pathology, University of Utah, Salt Lake City, UT 84108, USA; b Department of Pediatrics, Division of Medical Genetics, University of Utah, Salt Lake City, UT 84108, USA

**Keywords:** Amino acids, Prolidase deficiency, Glycyl-proline, Liquid chromatography-tandem mass spectrometry, Metabolomics

## Abstract

Prolidase deficiency (PD) is an autosomal recessive inborn error of metabolism, with an estimated incidence of 1 per 1.25 million births. Prolidase is critical for the turnover of proline and hydroxyproline-rich proteins, such as collagen. Collagen metabolism is essential for matrix regeneration during cellular proliferation and complex bodily functions such as wound healing and immunological cell differentiation. PD clinical manifestations include persistent skin ulcerations and poor wound healing, hypertelorism, high arched palate, depressed nasal bridge, micrognathia, splenomegaly, intellectual disability, recurring infections, and hematological abnormalities. Biochemically, a diagnosis of PD is supported by increased urinary excretion of glycyl-proline and other proline-containing iminopeptides detected by amino acid analysis. There are no current targeted therapies, but suggested interventions have included topical proline-glycine ointment, manganese supplementation, topical and oral steroids, and immunomodulation with monoclonal antibodies.

Here, we describe a 30-year-old patient with PD whose clinical course has been characterized by recurrent skin ulcerations/cysts with secondary scarring, recurrent infections, anemia, thrombocytopenia, lymphopenia, elevated liver enzymes, hirsutism, and seemingly unrelated papillary thyroid cancer. Skin manifestations were particularly severe due to the added complication of a diagnosis of hidradenitis suppurativa. Quantitative analysis of amino acids and related compounds revealed markedly elevated glycyl-proline in urine and plasma. To further characterize the biochemical phenotype, untargeted metabolomic analysis was sent on both plasma and urine. An increase was noted in several metabolites from the prolidase-dependent dipeptide recycling pathways. A better understanding of the pathophysiological mechanisms involved in prolidase deficiency may prove useful as different therapeutic approaches are being considered.

## 1 Introduction

**Fig. 1:**
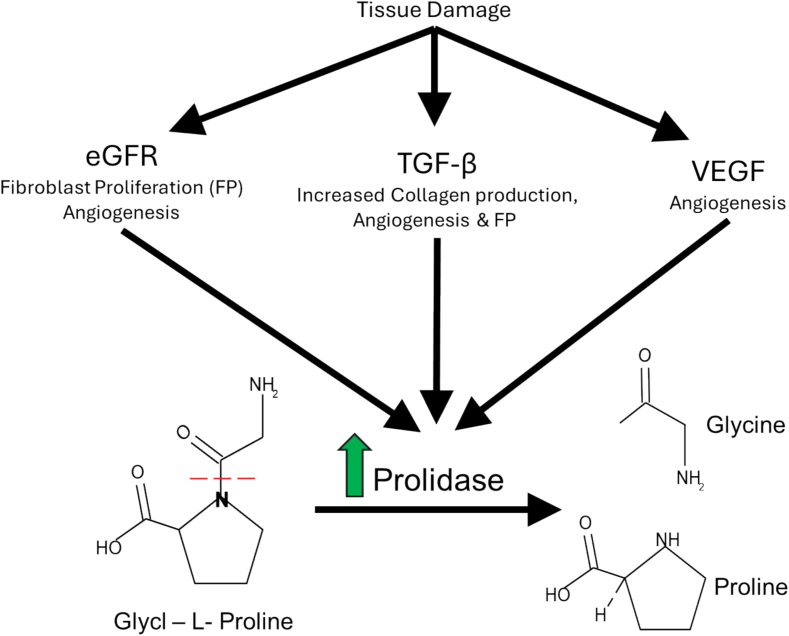
Tissue damage signals through TGF-β, eGFR, and VEGF to begin wound healing and reepithelization. Direct increase of PEPD transcription driven by TGF-β which in turn increases Prolidase enzyme activity. Prolidase catalyzes the cleavage of imidodipeptides, with a preference for Glycyl-L-Proline. The enzymatic cleavage cleaves glycl-proline on the dotted red line generating glycine and proline.

Prolidase deficiency (PD; OMIM#170100) is an extremely rare autosomal recessive inborn error of metabolism, with an estimated incidence of 1 per 1.25 million births [1]. The enzyme prolidase (EC 3.4.13.9), encoded by the *PEPD* gene, is the only known human dipeptidase that can hydrolyze the peptide bond preceding the amino acids proline or hydroxyproline [[2], [3], [4]]. Hence, prolidase is critical for the turnover of proline and hydroxyproline-rich proteins, such as collagen (Fig. 1). Collagen metabolism is essential for matrix regeneration during cellular proliferation and complex bodily functions such as wound healing and rapid immunological cell differentiation. Moreover, remodeling of collagen requires a pool of proline and hydroxylproline, which is supported by active prolidase enzyme activity [5].

The preferred substrate for collagen cleavage by the prolidase enzyme is the dipeptide Glycyl-L-Proline (Gly-Pro), but secondary substrates comprising the combination Xaa-Pro, where Xaa is any other proteogenic amino acid, can be utilized with varying impact on enzyme efficiency [6,7]. For normal enzyme function prolidase requires manganese as a metal ion cofactor [2,8]. With deficient prolidase activity, oligopeptides derived from collagen are not properly degraded, accumulate, and are excreted in urine [9]. The diagnostic dipeptide for prolidase deficiency is glycyl-proline, which is traditionally measured through urine amino acid analysis [8]. Although this dipeptide is the most specific biochemical marker for prolidase deficiency, other dipeptides such as alanine-proline (Ala-Pro), methionine-proline (Met-Pro), phenylalanine-proline (Phe-Pro), proline-proline (Pro-Pro), and several other can also be detected in smaller quantities [10].

The clinical manifestations of PD include persistent skin ulcerations and poor wound healing, hypertelorism, high arched palate, depressed nasal bridge, micrognathia, splenomegaly, variable degrees of intellectual disability, recurring infections, and hematological abnormalities the most common being thrombocytopenia (Spodenkiewicz, [1,11]). More recent studies associated with clinical analysis and interventions of a cohort of PD patients described chronic lung disease and systemic lupus erythematosus [1]. Biochemically, a diagnosis of PD is suggested by increased excretion of iminopeptides detected by urine amino acid analysis. When ion-exchange chromatography is used for amino acid analysis, several peaks are detected across the chromatogram corresponding to numerous Xaa-proline peptides [12]. Tandem mass spectrometry methods typically target the dipeptide Gly-Pro as biomarker for the condition; however, detection of multiple dipeptide species is possible and it has been reported [9].

Confirmation by *PEPD* gene sequencing has mostly replaced measurement of prolidase enzyme activity, due to limited availability of the testing [13]. Following a diagnosis, suggested therapies have included topical proline-glycine ointment, manganese supplementation, topical and oral steroid, and immunomodulation with monoclonal antibodies [14]. Here, we report the clinical and laboratory findings in a 30-year-old male patient with PD.

## 2 Case report

The patient was evaluated in childhood for developmental delays and diagnosed with PD at 5 years of age based on imidodipeptiduria, detected by ion-exchange chromatography. The diagnosis was confirmed by enzymatic assay, which reported a residual prolidase activity of 2 %. Following the diagnosis, the patient was lost to follow-up for several years. Family history was non-contributory; there are no other family members with a PD diagnosis and consanguinity was denied. Eventually, he was referred at 30 years of age to the University of Utah metabolic clinic by his primary care physician for evaluation. *PEPD* gene sequencing was ordered, which identified compound heterozygosity for a novel likely pathogenic variant, c.503 + 1G > C, disrupting an acceptor splice site in intron 6, and a previously published missense pathogenic variant c.826G > A (pAsp276Asn) [4]. c.826G > A; pAsp276Asn has been described in homozygosis in two unrelated PD patients that presented a similarly markedly reduced prolidase activity and severe phenotype [4]. c.503 + 1G > C is unreported but splicing variants affecting the same codon have also been associated to markedly reduced enzymatic activity and severe clinical findings [11].

At the time of the initial metabolic evaluation, the patient was observed to have typical facial features associated with PD such as hypertelorism, proptosis, and a slightly depressed nasal bridge. His clinical course since childhood had been characterized by recurrent skin ulcerations/cysts with secondary scarring, recurrent infections, asthma, joint laxity, hirsutism, mild anemia, thrombocytopenia, lymphopenia, mildly elevated liver enzymes, and seemingly unrelated papillary thyroid cancer. Thrombocytopenia, lymphopenia, and mildly elevated liver enzymes have been consistent throughout the recent course of his care. The skin manifestations were complicated by hidradenitis suppurativa, leading to painful subcutaneous cysts/abscesses progressing to open lesions and scarring. These had historically been managed with antibiotics, isotretinoin, frequent incision and drainage procedures, and hypochlorous acid. Table 1 summarizes the frequency of previously reviewed clinical findings in PD cases compared to our patient [1].

**Table 1:** Signs and symptoms in a cohort of PD patients (*n* = 197) reviewed by Rossingol et al. compared to our patient; number of patients with clinical manifestation and percent of total indicated. + indicates “present”, − indicates “not present”.

Clinical findings	Present/total	Percentage affected	Our patient
Ulcers	(122/197)	62 %	+
Other skin manifestations	(165/197)	84 %	+
Dysmorphic features	(132/197)	67 %	+
Chronic Respiratory	(53/197)	27 %	+
Hepato/Splenomegaly/Liver Dysfunction	(137/197)	70 %	+
GI Involvement	(26/197)	13 %	−
Hematologic Anomalies	(82/197)	42 %	+
Recurrent Infections	(99/197)	50 %	+
Immune Anomalies	(80/197)	41 %	+
Developmental Delay	(117/197)	59 %	+
Imidodipeptideuria	(139/197)	71 %	+

Two long-term disease-targeting therapies have been recommended by the metabolic clinic and followed with poor compliance by the patient. The first consisted of a collagen supplement which contained proline, glycine, hydroxyproline, at the suggested dose on the packaging. In addition, 500 mg of vitamin C was recommended to be taken daily. Both these supplements theoretically could support collagen synthesis and replace hydroxyproline, proline, and glycine that are less available in PD patients due to the inability to recycle. While trialing these supplements, adherence to recommended dosing was inconsistent and in the setting of an evolving cocktail of naturopathic supplements not managed by our clinic; due to these limitations, it is not clear whether there has been clear benefit from these interventions. Additionally, a topical intervention (tacrolimus cream) previously reported to help clear wounds in patients with PD [15] was recommended, but our patient only used it for a short time on hands and feet due to concerns for side effects.

Over the course of approximately 18 months, samples for quantitative analysis of amino acids in plasma and urine were collected in 5 different occasions. These samples were collected after clinic visits; no major changes were reported in the patient's health or skin findings between visits. He appeared and reported normal consistency of his disease and did not experience a major illness during our data collection. Amino acid analysis was performed using Liquid Chromatography-Tandem Mass Spectrometry (LC-MS/MS) and identified marked elevation of the disease-specific biomarker glycyl-proline in both plasma and urine. The analysis was performed at ARUP Laboratories as previously described [16]. Briefly, plasma and lyophilized urine samples were labeled with aTraq™ reagents (AB-Sciex) prior to analysis by an API 4000 tandem mass-spectrometer coupled with a SHIMADZU HPLC, operated in scheduled selective reaction monitoring mode (SRM) in positive mode. The specific transition monitored for glycyl-proline was 173.1 > 116.1; a six-point external calibration curve was used for quantitation. Lacking a commercially available internal standard (IS), taurine IS, which elutes at a similar retention time to the analyte, was used.

**Fig. 2:**
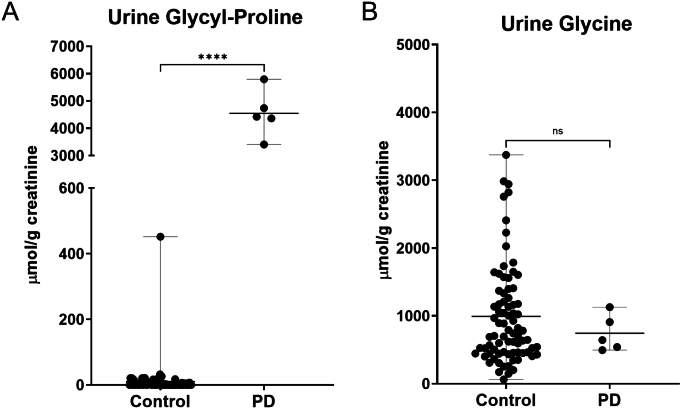
Urine glycyl-proline and glycine were quantified in the patient and compared to *n* = 79 age-matched clinical samples, interpreted as normal. PD = prolidase deficiency.

**Fig. 3:**
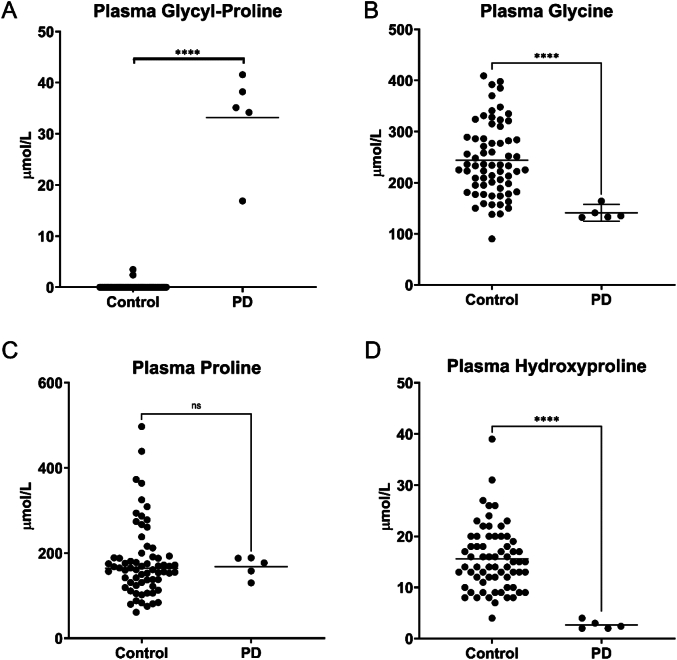
Plasma glycyl-proline, glycine, proline, and hydroxyproline were quantified in the patient and compared to *n* = 67 age-matched clinical samples, interpreted as normal. PD = prolidase deficiency.

Glycyl-proline was increased on average 100× times in urine and 30× in plasma compared to age-matched unaffected controls (Figs. 2A and 3A, respectively; *p* value <0.0001). Glycine was within the reference range in urine, and its median value in the PD patient's samples did not deviate when compared to the age-matched unaffected controls (*p* value = 0.768; Fig. 2B); moreover, we did not see changes between the values in the PD patient and unaffected controls for proline, hydroxyproline, and glycine in urine. In plasma, hydroxyproline (5–40 μmol/L reference interval) and glycine (140–420 μmol/L ref. interval) were below the lower limit of the reference range in most samples from our patient, with a lower median value compared to unaffected controls (*p* value <0.0001); while no statistically different change was seen for proline (90–350 μmol/L ref. interval) (Fig. 3B-D). In addition to amino acid analysis, the patient nutritional status was also assessed by evaluating essential fatty acids in plasma and red blood cells (RBC). Quantitation of long-chain fatty acids was performed as previously described [17,18] using gas chromatography-negative-chemical-ionization mass spectrometry (Agilent) following acid/base hydrolysis and derivatization. The results from both assays were essentially normal, although low concentration of several fatty acids was noted in the RBC, suggesting a low-fat diet. Furthermore, persistent thrombocytopenia and mildly elevated AST were noted by routine testing.

To better characterize the biochemical phenotype in this patient, untargeted metabolomic analysis (Global Metabolomic Assisted Pathway Screen, Global MAPS®) was sent on both plasma and urine to Baylor Genetics. Metabolites were identified based on the known chemical formula, nominal mass, and mass spectral fragmentation signatures with reference library entries created from authentic standard metabolites [19]. Metabolites were then organized in “super-pathways”, representing metabolite classes or general metabolic processes, and “sub-pathways” representing the specific metabolic pathways. Clinically relevant deviation from normal controls (z-score > 2 or < −2) were noted in urine for several metabolites from the prolidase-dependent dipeptide recycling pathways, including multiple hydroxyproline dipeptides (highlighted in Table 2). Interestingly, the dipeptide prolyl-glycine was not elevated (z score = 0.5); also, glycine, proline, and hydroxyproline did not have z-score values (>2 or < −2).

**Table 2:**
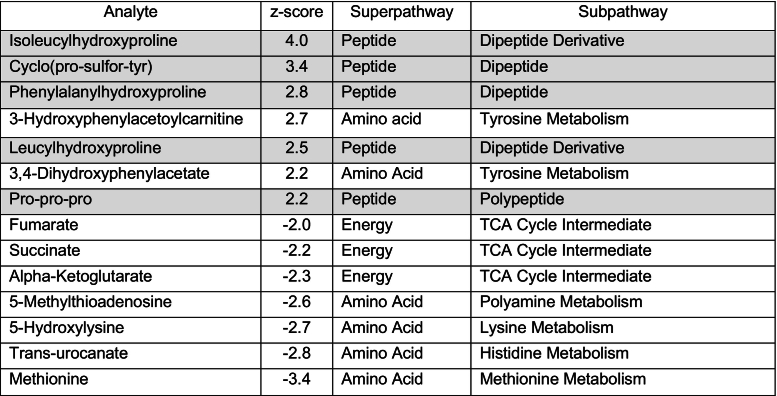
Urine metabolites with clinically relevant increases or decreases (z-score > 2 or < −2), organized in super- and sub-pathways using Baylor Genetics Global MAPS. Shaded in gray metabolites involved in the prolidase-dependent pathways.

In plasma, similar increases were seen for the peptide pro-hydroxy-pro; while trans-4-hydroxyproline, an hydroxyproline isomer, was decreased (highlighted in Table 3). Interestingly, we also observed modest changes in several other pathways, including lipid metabolism and TCA cycle metabolites; however, further data from PD patients is needed to assess the significance of these changes.

**Table 3:**
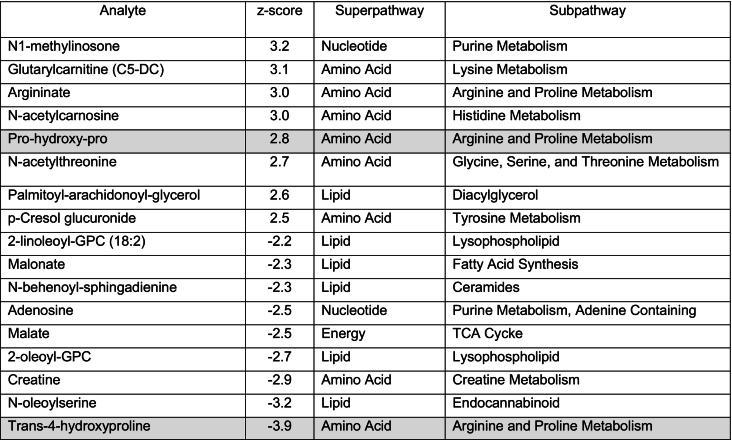
Plasma metabolites with clinically relevant increases or decreases (z-score > 2 or < −2), organized in super- and sub-pathways using Baylor Genetics Global MAPS. Shaded in gray metabolites involved in the prolidase-dependent pathways.

## 3 Discussion

In this case report, we have described the long-term follow-up of a patient with PD. Our patient's clinical characteristics were consistent with this condition, as described in the literature (Table 1). Interestingly, our patient had a separate diagnosis of hidradenitis suppurativa, which complicated his dermatological manifestations due to the presence of multiple draining abscesses that did not heal well and lead to worsened scarring. Additionally, outside of topical interventions (tacrolimus cream) or dietary supplementation with collagen supporting amino acid mixtures, no other interventions were pursued by the patient.

The diagnostic marker glycyl-proline was successfully detected by LC-MS/MS method both in urine and plasma. The inclusion of this analyte in LC-MS/MS-based methods for amino acid analysis is necessary to identify these patients. Outside of the marked increase in gly-pro, the urine and plasma profiles were essentially normal. However, further data from multiple individuals with PD is warranted to confirm our observations. Due to intermittent adherence to any proposed treatment, we cannot speculate on utilizing glycyl-proline to monitor therapeutic interventions. The variability that we observed in the analyte concentrations, in plasma more so than urine, likely reflect other variables such fasting status.

In this case report, we also confirmed that the “omics” level analysis is successful in identifying PD patients. To our knowledge, this is the first report that includes untargeted metabolomic data in a patient with this condition, providing further evidence that rare inborn errors of metabolism can be detected using metabolomics platforms. From our studies, we were able to identify a metabolite signature in PD that were not included in traditional LC-MS/MS methods and could be used in the future for detection of PD by metabolomics. Interestingly, metabolomic findings do not always replicate the same analyte abnormalities observed with traditional methods, emphasizing the importance of characterizing known patients with rare disorders with these new methodologies. We have also observed a mild decrease of TCA cycle metabolites in plasma (fumarate, succinate, alpha-ketoglutarate) and urine (malate) in our patient's metabolomic results (Table 2, Table 3). Anaplerosis of the TCA cycle through alpha-ketoglutarate has been previously shown to be reduced when proline is limited [20]. This decrease in TCA cycle metabolites could be an indication of inadequate proline availability due to the impaired prolidase activity.

Previously published studies on hidradenitis suppurativa skin biopsies showed metabolites associated to impaired tryptophan catabolism [21]. While analyzing the metabolomics data, we were unable to see clinically relevant changes in these metabolites in plasma and urine. However, no skin biopsies from our patient were available for the Global MAPS analysis; these tissues have been the ones predominantly used to study HS disorder [22].

In conclusion, we present our experience with a case of PD with classical clinical and targeted biochemical findings. Furthermore, we have expanded the known biochemical phenotype via untargeted metabolomic analysis to provide better understanding of the pathophysiological mechanisms in PD. Further characterization of these biomarkers may prove useful as different therapeutic approaches are being investigated.

## CRediT authorship contribution statement

**Troy K. Coody:** Writing – review & editing, Writing – original draft, Visualization, Data curation, Conceptualization. **Irene De Biase:** Writing – review & editing, Writing – original draft, Supervision, Conceptualization. **Julie M. Porter:** Writing – review & editing, Data curation. **Marzia Pasquali:** Writing – review & editing, Conceptualization. **Brian J. Shayota:** Writing – review & editing, Conceptualization.

## Funding sources

None.

## Declaration of competing interest

The authors declare no conflicts of interest with regard to the content of this report.

## Data Availability

Data will be made available on request.
